# γ-tubulin is differentially expressed in mitotic and non-mitotic cardiomyocytes in the regenerating zebrafish heart

**DOI:** 10.1016/j.dib.2015.01.005

**Published:** 2015-02-10

**Authors:** Pauline Sallin, Anna Jaźwińska

**Affiliations:** Department of Biology, University of Fribourg, Chemin du Musée 10, 1700 Fribourg, Switzerland

## Abstract

This data article contains complementary figures related to the research article entitled, “ A dual epimorphic and compensatory mode of heart regeneration” ([10], http://dx.doi.org/10.1016/j.ydbio.2014.12.002), which presents a spatial and temporal characterization of cardiomyocyte proliferation and dedifferentiation after cryoinjury-induced myocardial infarction. This study demonstrated that mitotic divisions occur in cardiac cells at distinct differentiation status, namely in dedifferentiated cells at the injury border as well as in mature cardiac cells within the remaining intact myocardium. One of the important aspects supporting our conclusions is a characterization of proteins that are upregulated during mitosis in the regenerating hearts. The data presented here reveal a dynamic change in the expression level and in the subcellular distribution of γ-tubulin between mitotic and non-mitotic cardiac cells. We report that in the non-mitotic cells, γ-tubulin expression is restricted to the centrosome. By contrast, during the mitosis, γ-tubulin strongly expands its localization within the spindle apparatus that interacts with the condensed chromosomes. We demonstrated that the differential distribution of γ-tubulin in non-mitotic and mitotic cells requires adjusted image processing for the appropriate visualization of both expression patterns in the same histological specimens.

**Specifications Table**

**Table:** 

Subject area	Biology

More specific subject area	*Regenerative biology*
Type of data	*Figure*
How data was acquired	*Confocal microscope (Leica Sp5)*
Data format	*Raw and processed with Adobe Photoshop*
Experimental factors	*Cryoinjuries were performed to induce myocardial infarction in transgenic adult zebrafish expressing EGFP under a cardiac specific promoter (cmlc2: EGFP). Hearts were collected at 14 days post cryoinjury, fixed in 2% paraformaldehyde, and sectioned using a cryostat.*
Experimental features	*Heart sections were analyzed using immunofluorescence against γ-tubulin, phospho-(Ser10)-histone H3, GFP and DAPI. The multiple labeling was analyzed using confocal microscopy. γ-tubulin fluorescent signals were adjusted for the optimal visualization of the subcellular distribution using the levels option of Adobe Photoshop software.*
Data source location	*University of Fribourg, Switzerland*
Data accessibility	*The data are supplied with this article*

**Value of the data**

• Quadruple immunolabeling with a cardiac transgenic marker, phospho-(Ser10)-histone H3, γ-tubulin and DAPI allows unambiguous identification of mitotic cardiomyocytes in the regenerating zebrafish heart.
• We analyze a previously uncharacterized distribution of γ-tubulin in the zebrafish adult cardiomyocytes to provide evidence of a differential expression pattern of γ-tubulin in non-mitotic and mitotic cardiac cells.
• We describe how to adjust the fluorescence signal intensity of the original confocal data in order to detect γ-tubulin either in the interphase centrosomes or in the mitotic spindle.

γ-tubulin is an evolutionary conserved cytoskeletal protein, which plays essential roles in microtubule organization and nucleation [4,6]. The subcellular distribution of this protein has been previously analyzed in a large variety of model organisms ranging from *Aspergillus* through *Drosophila* and mammals. In some of these studies, γ-tubulin was detected only in the centrosomes-related organelles during both the interphase and mitosis [7,11,12]. Other reports revealed the expansion of γ-tubulin during the mitosis within the nearly entire mitotic spindle [5]. To our knowledge, the distribution of γ-tubulin has not yet been analyzed in the zebrafish adult somatic cells. Previous studies revealed that the zebrafish heart regeneration depends on the proliferation of adult cardiomyocytes [8,9]. Here, to understand the mitotic mechanisms associated with zebrafish heart regeneration, we analyzed the expression of both γ-tubulin and phosphohistone H3 (PH3), which demarcates the condensed chromosomes during the nuclear division.

Our data provide evidence for the dynamic γ-tubulin expression during the cell cycle. To distinguish between the interphase/G0 and mitosis, we used phospho-(Ser10)-histone H3 (PH3) immunolabeling that demarcates the condensed chromosomes. To identify cardiomyocytes among other cell types in the heart, we used a transgenic fish line expressing EGFP under a cardiac specific promoter (*cmlc2*::*EGFP*), and we performed anti-GFP immunostaining (Fig. 1A-C; Fig. 3). Analysis of multiple heart sections revealed that in the non-mitotic zebrafish cardiomyocytes, γ-tubulin expression in restricted to a single spot in the vicinity of each nucleus, which corresponds to the centrosome [4]. By contrast, all of the PH3-positive cardiac cells were characterized by an expanded and stronger presence of γ-tubulin that was associated with the condensed chromosomes (*n*=17 cells, 5 hearts) (Fig. 1C and E; Fig. 3C and D). Analysis of the red fluorescence with the same image adjustments revealed that this centrosomal pattern of γ-tubulin expression does not derive from background enhancement (Fig. 2). Thus, γ-tubulin is not restricted only to the duplicated centrosomes of the dividing cells, but it covers other domains of the mitotic spindle. Due to the high difference in the intensity of γ-tubulin signals between mitotic and non-mitotic cells, it was not possible to simultaneously display both types of expression patterns on the same image. The original confocal data had to be adjusted using Adobe Photoshop to visualize both aspects of γ-tubulin expression in the separate images of the same original specimen (Fig. 1D and F; Fig. 3C′and D′). Our analyses of mitotic cytoskeletal proteins will be helpful to understand the cellular mechanisms underlying the proliferative capacity of adult zebrafish cardiomyocytes.

## 1 Experimental design, materials and methods

### 1.1 Animal procedures

The present work was performed with adult fish at the age of 18 months (transgenic fishes: *cmlc2*::*EGFP* zebrafish strains [1]. Cryoinjuries were performed as described previously [2,3]. The experimental research on animals was approved by the cantonal veterinary office of Fribourg.

### 1.2 Immunohistochemistry

The hearts were collected and fixed overnight at 4 °C in 2% paraformaldehyde. They were then rinsed in PBS and equilibrated in 30% sucrose before embedding in Tissue-Tek OCT compound (Sakura Finetek Europe B.V.) and cryosectioned at a thickness of 16 μm.

The immunohistochemistry procedure was performed as described previously [3]. The following primary antibodies were used: mouse anti-p-Histone H3 at 1:200 (Clone 3H10, Millipore), rabbit anti-tubulin-gamma (γ) at 1:2000 (Abcam, ab11321) and chicken anti-GFP at 1:2000 (Aves Labs, GFP-1010). The Alexa-Fluor-conjugated secondary antibodies (Jackson Immunoresearch) were used at 1:500, and DAPI was used at 1:2000. A detailed table describing the labeling and imaging settings for each structure is listed here:

**Table:** 

**Antigen**	**Primary antibody**	**Secondary antibody**	**Leica confocal excitation/emission filters (nm)**
**EGFP*(cmlc2*::*EGFP)***	chicken anti-GFP	anti-chicken alexa 488	488/ 500–540
***γ* -tubulin**	rabbit anti-tubulin-gamma	anti-rabbit Cy5	633/ 650–700
**PH3**	mouse anti-p-Histone H3	anti-mouse Cy3	543/ 560–600

### 1.3 Image analysis and quantification

After antibody staining, cardiac tissue imaging was performed at different magnifications (20× and 63×) with a confocal microscope (Leica TCS-SP5). The following Leica image acquisition parameters were used:

**Table:** 

**Magnification**	**20×**	**63×**
XY: Format (pixels)	1024×1024	1024×1024
XY: Speed (Hz)	200	200
Line average	3	3
Z-stacks (z-volume and number of steps)	2.5 μm; 3 steps	12.5 μm; 15 steps

The fluorescent pictures were then corrected using Adobe Photoshop for level adjustments.

The color balance and tonal range were optimized for each color channel by adjusting the midtones and highlights. For the green signal (γ-tubulin), the input level (image->adjustements->levels) was optimized to visualize either the spindle apparatus of mitotic cells (shadow (S): 0; midtones (M): 0.38; highlights (H): 255) or the centrosomes of non-mitotic cells (S: 0; M: 0.39; H: 44). The output levels were not changed.

## Figures and Tables

**Fig. 1:**
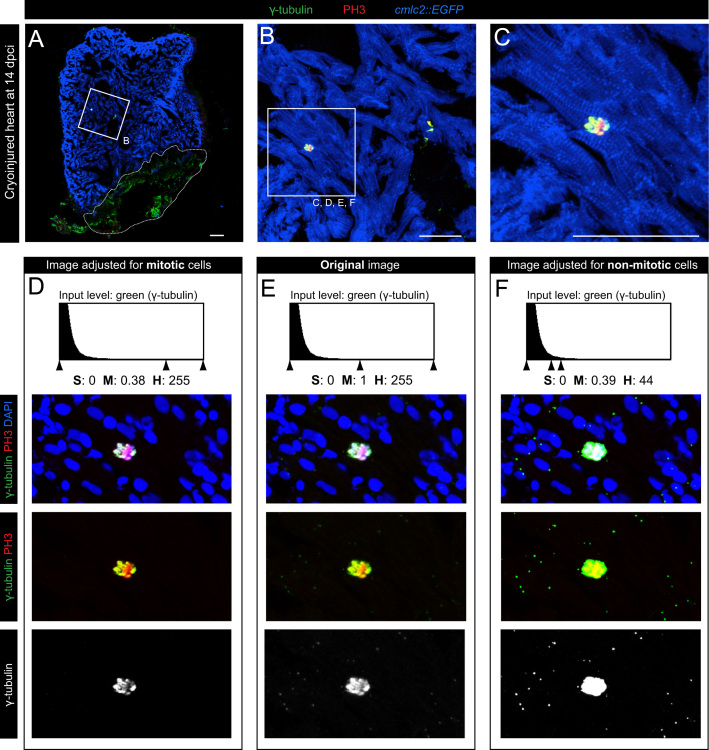
γ-tubulin is differentially distributed in mitotic and non-mitotic zebrafish cardiac cells. (A) Heart section of *cmlc2*::*EGFP* transgenic zebrafish at 14 days post cryoinjury (dpci) labeled with antibodies against GFP (*cmlc2: EGFP*, anti-GFP, cardiac cells, blue), phospho-(Ser10)-histone H3 (PH3, mitosis, red) and γ-tubulin (centrosomes, spindle apparatus, green). Cryoinjured part is encircled with a dashed line. (B, C) Higher magnification of the framed area shown in (A) showing a PH3-positive cardiomyocyte (C). (D–F) The same area as in (C) but contrastained with DAPI (blue), which colocalizes with PH3 (condensed chromosomes) and γ-tubulin immunolabeling. (E) Original confocal image. (D) The fluorescence signal of γ-tubulin was optimized to display the localization in the mitotic spindle. Using this setting, γ-tubulin expression in the centrosomes of the non-mitotic cells is undetectable. (F) Image adjustments according to the non-mitotic cell to detect the dotty pattern of centrosomal expression. Note, an overexposed γ-tubulin labeling of the mitotic cell. Scale bar (A, B, and C)=50 μm; S= shadows; M= midtones; H= highlights.

**Fig. 2:**
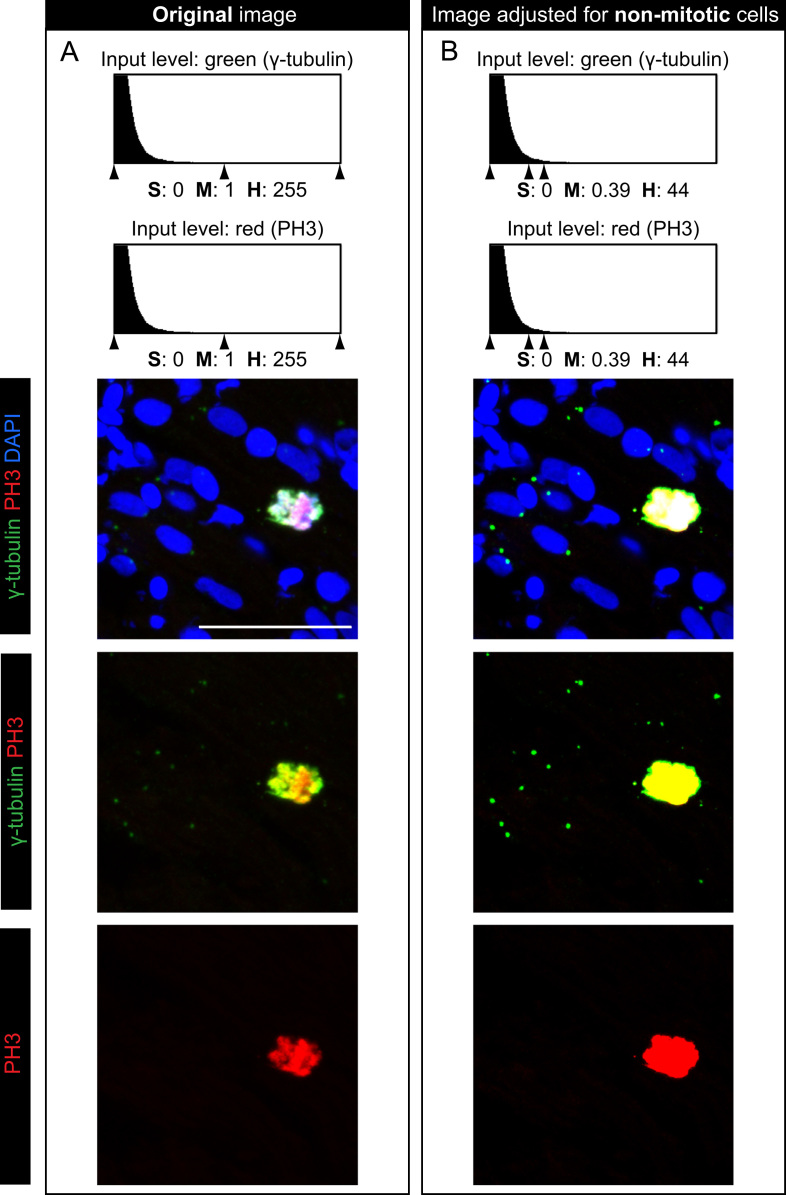
Dotty expression of γ-tubulin at the non-mitotic nuclei does not derive from enhanced background. (A) Higher magnification of the framed area shown in Fig.1 C labeled with PH3, γ-tubulin and DAPI. (B) The simultaneous increase of the green and red input levels reveals the dotty pattern of only γ-tubulin (green) but not PH3 (red). This demonstrates the specificity of green immunofluorescence consistent with the centrosomal localization of γ-tubulin. Scale bar (A)=25 μm; S=shadows; M=midtones; H=highlights.

**Fig. 3:**
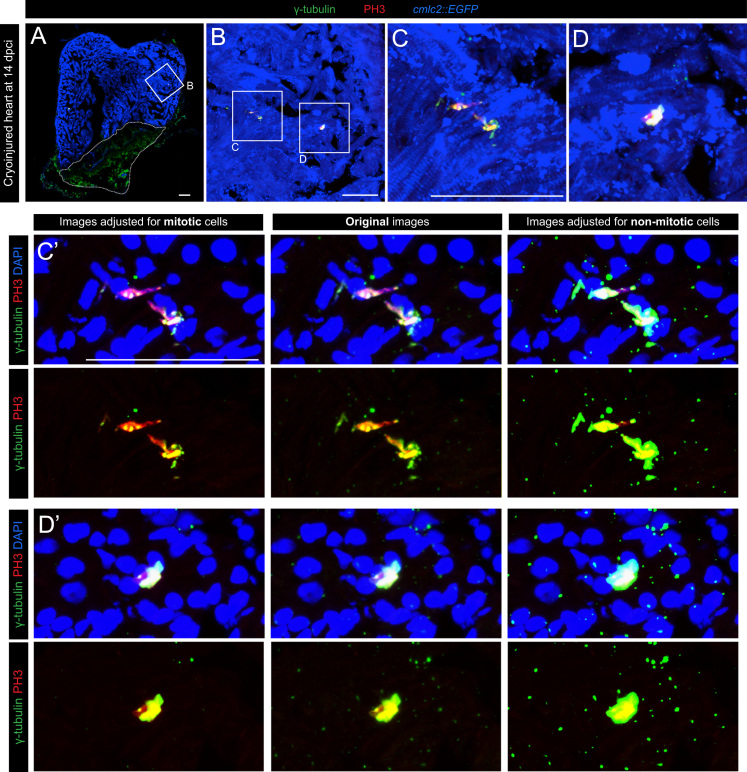
Additional examples of differential γ-tubulin expression in mitotic and non-mitotic cells. (A) Heart section of *cmlc2*::*EGFP* transgenic zebrafish at 14 days post cryoinjury (dpci) labeled with antibodies against GFP (*cmlc2*::*EGFP*, anti-GFP, cardiac cells, blue), phospho-(Ser10)-histone H3 (PH3, mitosis, red) and γ-tubulin (centrosomes, spindle apparatus, green). Cryoinjured part is encircled with a dashed line. (B) Higher magnification of the framed area shown in (A). (C, D) Higher magnification of the framed areas shown in (B) showing different PH3-positive cells. (C′, D**′**) The same areas as in (C) and (D) but contrastained with DAPI (blue). The differential subcellular expression of γ-tubulin in mitotic and non-mitotic cells was observed in several regions of each heart. *N*=5 hearts; Scale bar (A, B, C, C′)=50 μm; S=shadows; M= midtones; H=highlights.
